# Cost-effectiveness analysis of N95 respirators and medical masks to protect healthcare workers in China from respiratory infections

**DOI:** 10.1186/s12879-017-2564-9

**Published:** 2017-07-03

**Authors:** Shohini Mukerji, C. Raina MacIntyre, Holly Seale, Quanyi Wang, Peng Yang, Xiaoli Wang, Anthony T. Newall

**Affiliations:** 1 School of Public Health and Community Medicine, The University of New South Wales, Sydney, NSW 2052 Australia; 2 National Centre for Immunisation Research and Surveillance of Vaccine Preventable Diseases (NCIRS), University of Sydney, Westmead, NSW Australia; 3 The Beijing Centre for Disease Control and Prevention, Beijing, China

**Keywords:** Cost-effectiveness, Economic evaluation, N95 respirator, Mask, Healthcare worker

## Abstract

**Background:**

There are substantial differences between the costs of medical masks and N95 respirators. Cost-effectiveness analysis is required to assist decision-makers evaluating alternative healthcare worker (HCW) mask/respirator strategies. This study aims to compare the cost-effectiveness of N95 respirators and medical masks for protecting HCWs in Beijing, China.

**Methods:**

We developed a cost-effectiveness analysis model utilising efficacy and resource use data from two cluster randomised clinical trials assessing various mask/respirator strategies conducted in HCWs in Level 2 and 3 Beijing hospitals for the 2008–09 and 2009–10 influenza seasons. The main outcome measure was the incremental cost-effectiveness ratio (ICER) per clinical respiratory illness (CRI) case prevented. We used a societal perspective which included intervention costs, the healthcare costs of CRI in HCWs and absenteeism costs.

**Results:**

The incremental cost to prevent a CRI case with continuous use of N95 respirators when compared to medical masks ranged from US $490–$1230 (approx. 3000-7600 RMB). One-way sensitivity analysis indicated that the CRI attack rate and intervention effectiveness had the greatest impact on cost-effectiveness.

**Conclusions:**

The determination of cost-effectiveness for mask/respirator strategies will depend on the willingness to pay to prevent a CRI case in a HCW, which will vary between countries. In the case of a highly pathogenic pandemic, respirator use in HCWs would likely be a cost-effective intervention.

## Background

Healthcare workers (HCWs) are at increased risk of contracting influenza and other respiratory infections compared to the rest of the adult working population [1]. The use of respirators and masks to reduce transmission of these infections within hospitals can decrease the costs associated with HCW absenteeism and the costs of nosocomial infections in vulnerable patients. Mask/respirator availability can also be crucial in the context of emerging respiratory infection threats to HCWs where effective pharmaceutical interventions may not be available, e.g. the 2003 severe acute respiratory syndrome (SARS) epidemic and the Middle East Respiratory Syndrome (MERS) outbreaks since 2012 [2, 3].

Medical masks, referred to as surgical masks in some countries [4], were not designed to protect the wearer from aerosol transmission of droplet nuclei and viral particles [5, 6]. Respirators are specially engineered for this purpose [7]. The N95 respirator models have been tested and proven to have at least 95% particle filter efficiency [8]. The World Health Organization (WHO) and the U.S. Centres for Disease Control and Prevention (CDC) recommend the use of a mask in low-risk settings and a respirator in high-risk settings (e.g. during aerosol generating procedures) to protect healthcare workers (HCWs) from seasonal influenza [9, 10]. In the context of influenza A(H1N1)pdm09, the WHO recommended similar precautions to those advised for seasonal influenza while the CDC recommended at least the equivalent of a fit tested N95 respirator for HCWs in contact with pandemic influenza patients, even when aerosol-generating procedures were not being conducted [11, 12].

There are substantial differences between the costs of respirators and masks that may affect the development of country specific mask/respirator guidelines for HCWs [4]. Another potentially important consideration is fit testing, which adds to the cost of respirator use, but may help ensure the seal of a respirator to a HCW’s face [7]. A recent review identified the lack of economic evaluations that used clinical efficacy estimates to assess the cost-effectiveness of mask/respirator use in healthcare settings [13]. The aim of this present study is to compare the cost-effectiveness of respirator and mask interventions in HCWs using data from two large clinical trials conducted in Beijing hospitals [14, 15].

## Methods

### Trial and intervention design

The two previously published cluster randomised trials used in this economic evaluation [14, 15] were designed to measure the efficacy of N95 respirators and medical masks to protect HCWs from respiratory infections in Beijing hospitals. In these trial designs, the units of randomisation were entire emergency departments or respiratory wards from Level 2 and 3 hospitals, assigned to each intervention. The participants were the HCWs working in each of the enrolled wards, i.e., all nurses, doctors and administration staff. These wards were selected as they were considered to be high-risk settings for occupational exposure to respiratory infections. The intervention period in the first trial (Trial 1) was during the winter of 2008/09, from December 2008 to January 2009 [14]. The second trial (Trial 2) intervention period followed in the winter of 2009/10, from December 2009 to January 2010 [15].

In Trial 1 continuous use of fit tested and non-fit tested N95 respirators was compared to medical masks [14]. Continuous use referred to the wearing of N95 respirators or medical masks for the entire shift. It was deemed unethical to randomise wards to a control arm (i.e. no mask use), so a convenience sample of HCWs from hospitals with documented pre-study low rates of mask use was recruited. This arm was excluded from this economic analysis due to the lack of randomisation. In Trial 2 there were two fit tested N95 respirator arms and the difference between these arms was that one arm employed continuous respirator use while the other carried out targeted (i.e. selective) respirator use by HCWs only whilst conducting high-risk procedures such as common aerosol-generating procedures, or when barrier nursing patients with known respiratory illness. These arms were compared to a continuous use medical mask arm (as used in Trial 1).

In Trial 1, the number of HCWs per arm was 492, 461 and 488 for the continuous medical mask, continuous N95 fit tested and continuous N95 non-fit tested arms respectively. In Trial 2, the number of HCWs per arm was 572, 516 and 581 for the continuous medical mask, targeted N95 fit tested and continuous N95 fit tested arms respectively. The criterion for clinical respiratory illness (CRI) in both trials was the presentation of two or more respiratory symptoms or one respiratory symptom and one systemic symptom [14, 16]. Respiratory symptoms considered were cough, nasal congestion, rhinorrhoea, sore throat and sneezing. Systemic symptoms considered were a temperature > 38 °C, perceived chills and/or fever, lethargy, loss of appetite and myalgia.

The respirators and masks used in both trials were sourced from the manufacturer 3 M China [14, 15]. As the models supplied in Trial 1 had been discontinued for China by the following season, different models of the same respirator and mask types were supplied in Trial 2. Both trials included at least one arm that applied qualitative fit testing for participating HCWs once at the beginning of the trial period, according to the manufacturer’s instructions [17].

### Perspectives

We adopted a societal perspective, as recommended by the WHO cost-effectiveness analysis guidelines [18]. We included the costs for the interventions in addition to inpatient and outpatient costs to the healthcare system for HCW CRI cases. Indirect (productivity) costs were included for 15 min of staff time lost while being fit tested for respirators and due to HCW absenteeism for CRI. The only out-of-pocket costs included were the costs of medications and outpatient visits to healthcare centres/fever clinics and emergency wards for CRI. Other potential out-of-pocket costs incurred to HCWs with CRI, such as healthcare co-payments and direct non-medical costs (e.g. transport costs) were excluded as no trial data was collected on these items.

### Economic model design

We applied a decision analytic model using Microsoft Excel to construct a separate economic evaluation for each trial. Costs and health outcomes were not discounted as the time horizon of the analysis was less than one year (28 days). The primary outcome was the incremental cost effectiveness ratio (ICER) per CRI case prevented. This was chosen as CRI in HCWs was the primary endpoint in both trials (CRI cases in patients were not recorded). A one-way sensitivity analysis was carried out on a range of parameters to identify those that most substantially impacted on the ICER. In scenario analysis we examined seasonal variation in attack rates for CRI (from 1–30%) [1] to capture values outside of those observed in the years the trials were run.

Before adjusting for confounding, Trial 1 found that only the continuous use non-fit tested N95 arm was significantly more protective against CRI than medical masks [14]. However, a combined ‘All N95’ arm, which included both fit tested and non-fit tested N95 groups (there was no significant difference between these groups), was found to be significantly more protective in post-hoc cluster confounder adjusted analysis compared to medical masks [14]. For these reasons, we calculated our ICER results for this trial using the efficacy estimated for this ‘All N95’ arm. However, to explore the impact of fit testing on cost-effectiveness, we selectively either included/excluded the cost of fit testing for those in the ‘All N95’ arm. For Trial 2, after the results were cluster and confounder adjusted, only the continuous use fit tested N95 arm had a significantly more protective effect compared to medical masks [15]. We excluded the targeted use fit tested N95 arm from the economic analysis as there was no significant difference in results for this arm when compared to medical masks [15].

### Parameter input sources

The CRI rates observed in each of the arms of the trials were used when reporting preliminary results for all arms, however when estimating ICERs, CRI rates were generated from the intention-to-treat cluster and confounder adjusted significant efficacy results from both trials [14, 15]. Information on intervention efficacy, the number of respirators or masks used per shift and shift durations, were obtained from the trial publications [14, 15]. Data on HCW resource use due to CRI cases, the exact proportions of each staff type in each arm, the number of shifts worked and the number of days of leave taken, were extracted from the trial databases. Healthcare resource use costs for treatments were sourced from the Beijing pharmaceutical sunshine procurement platform [19]. Estimates were used for the costs of visits to healthcare centres or emergency departments and staff monthly salary costs (Xiaoli Wang, Beijing CDC, personal communication, July 2014). The estimated salary costs were comparable (approximately) with the National Bureau of Statistics China average health and social work salary for 2014 [20]. Costs are reported in 2014 US Dollars and 2014 Chinese Renminbi using the exchange rate of 1 USD = 6.2RMB (Table 1).

**Table 1:** Unit costs associated with intervention strategies in Beijing hospitals for the 2008–09 and 2009–10 influenza seasons

Parameters	Base case value (2014 USD, Chinese RMB)	Source
Equipment costs (‘list price’ per unit)^a^		
Medical mask	0.14 (1)	3 M China, 3 M Standard Tie-On Surgical Mask (catalogue number mask 1817)^b^
N95 respirator	0.79 (5)	3 M China, 3 M flat-fold N95 respirator (catalogue number 9132)^c^
Fit Test Kit	608 (3770)	3 M China, 3 M FT-30 Bitrex Fit Test Kit^c^
Productivity cost of HCW time to be fit tested^d^		
Doctor	2.48 (15) Trial 1 2.26 (14) Trial 2	Calculated based on estimated monthly staff salaries
Nurse	1.74 (11) Trial 1 1.58 (10) Trial 2	Calculated based on estimated monthly staff salaries
Administration staff	1.98 (12) Trial 1 1.81 (11) Trial 2	Calculated based on estimated monthly staff salaries
Unit costs associated clinical respiratory illness (CRI)		
Direct costs		
Antibiotics (e.g. Azithromycin 6 tablets)	1.93 (12)	Beijing pharmaceutical sunshine procurement platform [19]
Antitussives (e.g. Apricot cough syrup 250 ml bottle)	4.03 (25)	Beijing pharmaceutical sunshine procurement platform [19]
Antipyretics (e.g. Paracetamol 12 tablets)	1.45 (9)	Beijing pharmaceutical sunshine procurement platform [19]
Antivirals (e.g. Oseltamivir 10 tablets)	35.65 (221)	Beijing pharmaceutical sunshine procurement platform [19]
Traditional. Chinese Medicine (e.g. Ganmao Qingre Granules) 10 bags	1.93 (12)	Beijing pharmaceutical sunshine procurement platform [19]
Healthcare centre/fever clinic/hospital outpatient visit^f^	8.06 (50)	Estimate^e^
Emergency ward visit^f^	16.12 (100)	Estimate^e^
Monthly staff salaries^e^		
Doctor	1613 (10000)	Estimate^g^
Nurse	1129 (7000)	Estimate^g^
Administration	1290 (8000)	Estimate^g^

^a^The ‘list price’ costs for large, health based orders within China (in its economic and taxation framework)

^b^(Terry Gorman, 3 M Senior Occupational Hygienist, personal communication, January 2012). The 2012 cost for medical mask was used as no updated cost was made available at the time of enquire

^c^(Terry Gorman, 3 M Senior Occupational Hygienist, personal communication, September 2014)

^d^Staff time productivity costs for an estimated 15 min of fit testing (based on monthly salary) differs between Trial 1 and 2 due to the slightly greater number of shifts worked per month by HCWs in Trial 2

^e^(Xiaoli Wang, Beijing CDC, personal communication, July 2014)

^f^Estimated costs for Beijing level 2 hospitalisations were applied in the model

^g^Salary estimates were comparable (approximately) with the average Beijing health and social work salary for 2014 [20]

### Costs of intervention

For both trials separately, respirator and mask costs per HCW for each arm were calculated by multiplying the mean number of shifts worked in the 28 day trial periods, by the cost of either two N95 respirators or three medical masks as provided per HCW for each shift worked in the trials [14, 15]. HCWs worked an average of 20 shifts in Trial 1 and 22 shifts in Trial 2 over the trial duration, with a typical shift being eight hours in duration.

For fit tested arms, the mean cost of 15 min of HCW time to be fit tested was included, taking into account the proportion of each staff type (doctors, nurses, etc.) in the trial. Fit tester time was included as the cost of 15 min of an administration staff member’s time per HCW, US $1.98 (12 RMB) in Trial 1 and US $1.81 (11 RMB) in Trial 2. This cost (calculated from monthly salaries) was estimated to be different between the trials as HCWs worked slightly more shifts per month on average in Trial 2. As test operators can be self-taught we did not include any training costs [17]. The respirator, mask and fit test kit costs applied are shown in Table 1 (models from 3 M China: 3 M flat-fold N95 respirator [catalogue number 9132], 3 M Standard Tie-On Surgical Mask [catalogue number mask 1817], and 3 M FT-30 Bitrex Fit Test Kit). The mean cost of a fit test kit applied to each HCW was calculated by multiplying the number of wards in the arm by the cost of a kit, and dividing this by the total number of HCWs in that respective arm. This simulated the likely occurrence that in practice one fit test kit would be purchased for each hospital ward conducting fit testing (Xiaoli Wang, Beijing CDC, personal communication, July 2014).

### Cost of CRI

Information on the direct healthcare resource use of each HCW who was documented as having a CRI during the trials was collected through surveys completed in person. HCWs who had symptoms were required to report to the ward head nurse for survey completion. This provided data on the different types of treatment used by the HCW for a specific CRI episode and on the number of healthcare centre/fever clinic visits and emergency ward visits. This was used to calculate the mean cost of CRI for a HCW in each trial.

Indirect costs, in terms of productivity loss due to HCWs taking leave for a CRI episode, were estimated using the human capital approach [21]. The mean leave cost per HCW with CRI in each trial was calculated taking into account the proportions of each staff type that took leave. The productivity cost per day of leave was calculated by dividing mean monthly income by the average number of shifts for all HCWs in the 28 days trial period (i.e. 20 shifts in Trial 1 and 22 in Trial 2).

### CRI rates

In Trial 1 the CRI attack rates for those allocated to continuous N95 use were 4.6% and 3.3% (with/without fit testing respectively) and 6.7% for those allocated to the medical mask use arm (Table 2) [14]. In Trial 2, the CRI attack rate was 7.2% for those allocated to fit tested N95 respirators compared to 17.1% for those allocated to the medical mask use arm (Table 2) [15]. These were also compared to a CRI attack rate of 11.8% for the targeted use fit tested N95 arm in Trial 2 [15], which was excluded from further analysis due to lack of statistical difference compared to the medical mask arm.

**Table 2:** Clinical respiratory illness (CRI) and average costs per HCW in each intervention arm for Beijing Trial 1 (2008/09) and Trial 2 (2009/10)

Clinical respiratory illness (CRI) and average costs per HCW in each intervention arm in Trial 1 (Beijing 2008/09) with the adjusted estimate for All N95 combined shown in bold					
	Continuous medical mask, 2014 USD, (RMB)	Continuous N95 non-fit tested 2014 USD, (RMB)	Continuous N95 fit tested 2014 USD, (RMB)	**Continuous All N95 (without fit testing cost)** **2014 USD, (RMB)**	**Continuous All N95 (with fit testing cost)** **2014 USD, (RMB)**
CRI %	6.7%	3.3%	4.6%	**2.6%** ^**a**^	**2.6%** ^**a**^
Estimated cases prevented per 1000 HCWs compared to medical mask	NA	34	22	**41** ^**a**^	**41** ^**a**^
Intervention costs per HCW	8.53 (53)	32.07 (199)	60.40 (374)	**32.07 (199)**	**60.40 (374)**
CRI costs per HCW	0.81 (5)	0.39 (2)	0.64 (4)	**0.31 (2)**	**0.31 (2)**
Total costs per HCW	9.34 (58)	32.46 (201)	61.04 (378)	**32.48 (201)**	**60.72 (376)**
Clinical respiratory illness (CRI) and average costs per HCW in each intervention arm in Trial 2 (Beijing 2009/10) with the adjusted estimate for continuous N95 fit tested shown in bold					
	Continuous medical mask 2014 USD, (RMB)	Targeted N95 fit tested 2014 USD, (RMB)	Continuous N95 fit tested 2014 USD, (RMB)	**Continuous N95 fit tested** **2014 USD, (RMB)**	
CRI %	17.1%	11.8%	7.2%	**6.7%** ^**b**^	
Estimated cases prevented per 1000 HCWs compared to medical mask	NA	53	99	**105** ^**b**^	
Intervention costs per HCW	9.35 (58)	61.31 (380)	61.31 (380)	**61.31 (380)**	
CRI costs per HCW	1.40 (9)	0.97 (6)	0.59 (4)	**0.55 (3)**	
Total costs per HCW	10.75 (67)	62.28 (386)	61.90 (384)	**61.86 (384)**	

^a^Results calculated based on the clustering and confounder adjusted odds ratio (0.38) for All N95 compared to medical mask (equivalent to a 62% efficacy = 1 - OR)

^b^Results calculated based on the clustering and confounder adjusted hazard ratio (0.39) for continuous N95 fit tested compared to medical mask (equivalent to a 61% efficacy = 1 - HR)

## Results

### Estimated mean intervention costs

Intervention costs per HCW in Trial 1 for the 28 days were US $32.07 (199 RMB) in the continuous use non-fit tested N95 arm. They were nearly two-fold higher for the continuous use N95 fit tested arm at US $60.40 (374 RMB) and were US$8.53 (53 RMB) for the medical mask arm (Table 2). Results for Trial 2 were similar, with intervention costs per HCW of US$61.31 (380RMB) for both N95 arms and US $9.35 (58 RMB) per HCW for the medical mask use arm (Table 2).

### Estimated mean CRI costs

**Fig. 1:**
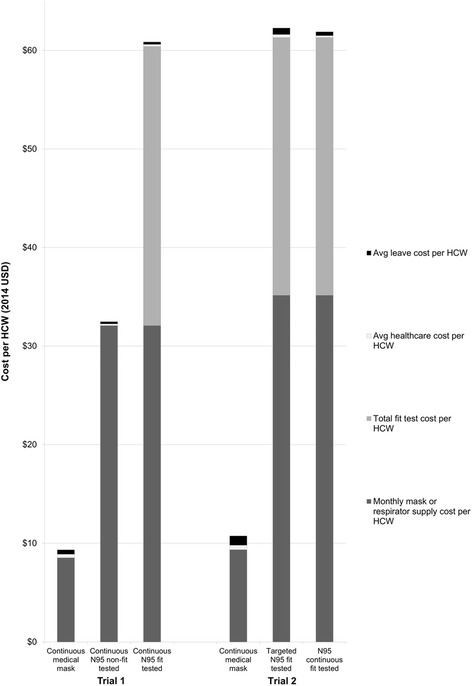
Average costs per healthcare worker (HCW) in Beijing Trial 1 (2008/09) and Trial 2 (2009/10). The shaded sections within each bar represent different components of the average intervention and treatment costs per HCW for the 28 day period in each trial arm

The mean CRI costs per HCW case were low in all arms compared to intervention costs (Table 2). The most commonly reported treatment used by those with CRI in both trials (56% in Trial 1 and 30.5% in Trial 2) was traditional Chinese medicines (commonly Ganmo Qingre Granules, US $1.93 [12 RMB]). A breakdown of mean CRI costs per HCW and mean intervention costs per HCW for respective trial intervention arms is shown in Fig. 1.

Mean direct healthcare costs per HCW with CRI in Trial 1 were higher (US $5.45 [34 RMB]) than in Trial 2 (US $2.60 [16 RMB]). Mean indirect costs per HCW with CRI were similar between the trials at US $6.53 (40 RMB) in Trial 1 and US $5.56 (34 RMB) in Trial 2.

### ICER

The cost-effectiveness estimates described below are focused around the intention-to-treat trial efficacy results for N95 respirators which showed a significant protective effect against CRI, when compared to medical masks, after adjusting for clustering and confounding (shown in bold in Table 2).

### Continuous use of fit tested N95 respirators vs. medical masks

In Trial 1, when we included the cost of fit testing, the ICER for continuous N95 respirator use was US $1224 (7589 RMB) per CRI case prevented when compared to medical mask use. In Trial 2 the ICER was US $489 (3032 RMB) for continuous fit tested N95 respirator use when compared to medical mask use. The difference between the trials is primarily explained by the higher CRI attack rates in Trial 2 (Table 2).

### Continuous use of non-fit tested N95 respirators vs. medical masks

In Trial 1, when we excluded the cost of fit testing, the ICER for continuous N95 respirator use was US $549 (3404 RMB) per CRI case prevented when compared to medical mask use. In response to the relatively low fit test failures observed in both trials of 1.1–2.6% [14, 15], we also estimated the ICER for Trial 2 under a hypothetical scenario where the cost of fit testing was excluded from the respirator arm. This scenario analysis resulted in an ICER for continuous N95 use of US $239 (1482 RMB) per CRI case prevented compared to medical masks.

### Sensitivity analysis results

**Fig. 2:**
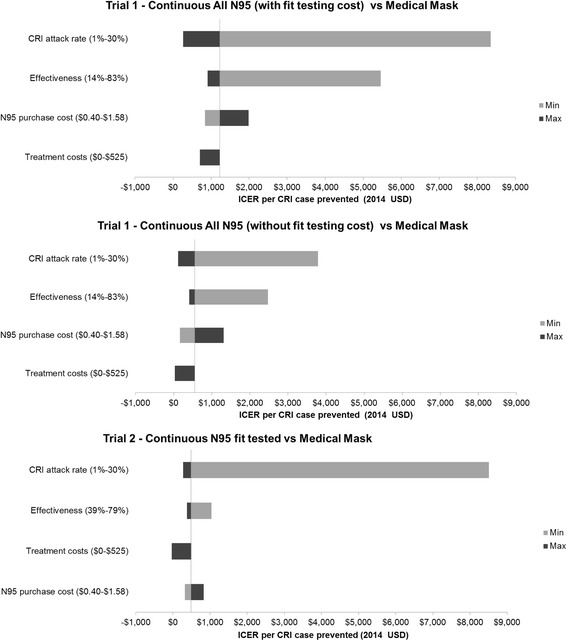
One-way sensitivity analysis of key parameters on the incremental cost-effectiveness ratio (ICER) per CRI case prevented in Beijing Trial 1 (2008/09) and Trial 2 (2009/10)

**Fig. 3:**
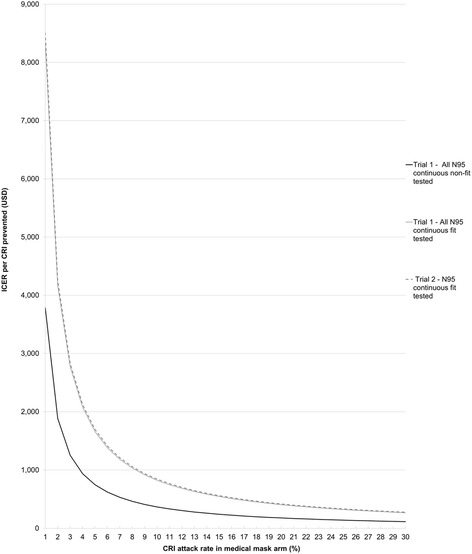
Sensitivity analysis on the incremental cost-effectiveness ratio (ICER) based on variation in the attack rate in the medical mask arm. Variation in the clinical respiratory illness (CRI) attack rates in this arm represent potential seasonal differences in transmission. Results are for Beijing Trial 1 (2008/09) and Trial 2 (2009/10). Note: the lines for Trial 1 – All N95 continuous fit tested and Trial 2 – N95 continuous fit tested overlap

One-way sensitivity analysis indicated that CRI attack rate and intervention effectiveness have the greatest impact on the ICERs (Fig. 2). In scenarios where we assumed the attack rate was greater than approximately 4% for continuous non-fit tested respirators and 8% for fit tested respirators, the ICERs were below US $1000 (6200 RMB) per CRI case prevented (see Fig. 3). The importance of the attack rate is also shown by the overlap in the Trial 1 and 2 results for the N95 continuous fit tested arms when we modelled the same background CRI attack rate in the medical mask arm (see Fig. 3).

In both trials the ICER worsened substantially when the lower effectiveness values from the trial confidence intervals were applied (see Table 3 and Fig. 2). Sensitivity analysis was also carried out on costs for treatment including healthcare visits and the impact of doubling and halving the cost of N95 respirators (see Table 3 and Fig. 2). The potential for the interventions to be cost-saving was only estimated when we assumed a severe illness treatment cost for each CRI case (US $525, 3255 RMB) [22]. This is an extreme scenario and is unlikely except in a highly pathogenic influenza epidemic/pandemic where a mean case requires substantial healthcare treatment.

**Table 3:** Sensitivity analysis on the ICER based on variation in intervention efficacy and costs for Beijing Trial 1 (2008/09) and Trial 2 (2009/10)

Parameter input value		Trial 1		Trial 2	Source
		Continuous All N95 (without fit testing cost) 2014 USD, (RMB)	Continuous All N95 (with fit testing cost) 2014 USD, (RMB)	Continuous N95 fit tested 2014 USD, (RMB)	
Base case results	-	549 (3404)	1224 (7589)	489 (3032)	-
Intervention efficacy vs. Medical Mask					
Lowest effectiveness^a^	14% (Trial 1) 39% (Trial 2)	2473 (15333)	5462 (33864)	1038 (6436)	[14] [15]
Highest effectiveness^a^	83% (Trial 1) 79% (Trial 2)	407 (2523)	911 (5648)	376 (2331)	[14] [15]
Alternative costs					
Max treatment costs	$525^b^ (3255 RMB)	30 (186)	704 (4365)	Cost saving^b^	[22]
Min treatment costs	$0 (0 RMB)	555 (3441)	1230 (7626)	492 (3050)	Assumption
N95 purchase cost doubled	$1.58 (8 RMB)	1313 (8141)	1988 (12326)	825 (5115)	Assumption
N95 purchase cost halved	$0.40 (2 RMB)	167 (1035)	842 (5220)	321 (1984)	Assumption

^a^High and low efficacy estimates calculated from the confidence intervals generated for the clustering and confounder adjusted results from Trial 1 and 2 respectively

^b^This severe illness treatment cost for each CRI case [22] is unlikely except in a highly pathogenic influenza epidemic/pandemic where an average case requires substantial healthcare treatment

## Discussion

We estimated that the incremental cost to prevent a CRI case in a HCW for continuous N95 respirator interventions compared to medical masks ranged from US $490–$1230 (3000-7600 RMB) in this setting, which is in the acceptable range. We found that including fit testing in a N95 respirator intervention approximately doubles the cost of the intervention and substantially decreases the cost-effectiveness per CRI case prevented compared to medical masks during seasons with a low CRI attack rate. However, these results must be interpreted with caution as the low respirator fit test failure rates (1.1–2.6%) observed in the trials may be specific to the N95 respirator models used in both trials [14, 15]. A potential policy option could be to forego fit testing if respirators with known low fit test failure rates were used (such as those used in the trials), although this decision requires careful consideration and would depend on the severity of circulating pathogens. This incremental approach to considering the additional costs and benefits of fit testing may be particularly important in settings where resources are limited and choices must be made between fit testing and other potential lifesaving interventions. At present there is insufficient literature to support that the low failure rates seen in the trials would apply for all respirator models.

We also found that variation in the CRI attack rate was a major factor in determining the cost-effectiveness of respirators (see Fig. 2). The higher attack rate in Trial 2 was most likely due to the more active influenza season observed in the 2009/10 influenza season [14, 15]. The incidence and severity of CRI cases which occur in any given year will vary in accordance with the transmissibility and pathogenicity of the annual influenza strains that are circulating during that season. The incremental cost per CRI case prevented for continuous use N95 respirators compared to medical masks was found to be substantially lower in high attack rate seasons (Fig. 3). It is important that this seasonal variation is accounted for when evaluating the cost-effectiveness of influenza prevention measures [23].

Whilst the results of our study are indicative of cost-effectiveness, economic evaluations are not usually seen as transferable between settings. Mask/respirator interventions in HCWs need to be evaluated for countries separately to account for inter-country variations in factors such as intervention acceptability [24, 25], healthcare costs and productivity costs. Variation within countries is also possible and hence the results of this study which only includes trial data from Beijing hospitals may not be generalisable to all settings in China.

China has comparatively high cultural acceptability of mask/respirator use compared to western countries [26]. Furthermore, a study of Beijing HCWs regarding A(H1N1)pdm09, suggests that some HCWs in this setting may continue to attend work with symptoms of influenza like illness [27]. This may partially explain the relatively low levels of absenteeism for HCWs with CRI in the trials. The low mean healthcare costs for CRI in both trials may have been due to the relatively modest influenza seasons and low levels of healthcare seeking by staff [14, 15]. The existing economic studies on masks/respirators have suggested that the interventions were likely to be cost-effective in the high income settings they examined [28–32]. However, it is difficult to compare the results of our evaluation to these previous high income setting studies as they often did not report results in an easily comparable format (e.g. cost per case prevented) and most did not use clinical trial efficacy data to inform their analyses [33].

A limitation of the original trials was the 28 day duration [14, 15] meaning that the results may not translate to longer term interventions which may have different adherence levels. A limitation of our analysis is that the effect of the interventions on CRI rates in the patients or family members of HCWs could not be included due to the absence of data. The exclusion of these factors will make our results more conservative. HCWs are known to transmit various types of nosocomial infections to patients [34] and the literature indicates that there are likely to be substantial benefits if the number of patients with CRI is reduced [22]. For example, the benefits of preventing HCW infection transmission through vaccination have been observed in long-term care facilities that found an associated decrease in mortality in their elderly residents [35–37]. The difference in mean direct healthcare costs per HCW with CRI between the trials (i.e. US $5.45 [34 RMB] in Trial 1 and US $2.60 [16 RMB] in Trial 2) may partly reflect variation in the thoroughness of the sick follow-up data collection between the trials. Another potential limitation of this study is that stochastic uncertainty was not directly explored using the individual level data from the trials [21]. Instead, we adopted a decision-model based approach using clinical trial data to inform input parameter values and explored the uncertainty of these values in deterministic sensitivity analyses. This approach facilitated a focus on forms of uncertainty unrelated to sampling, such as the potential inter-year variation in the CRI attack rate. Finally, as data on quality of life were not collected in the trial we chose to focus the economic analysis on the primary trial outcome (CRI). This limits the ability to compare the value for money offered against other interventions.

## Conclusions

This economic evaluation is the first economic analysis of mask/respirator interventions to be conducted for a middle income setting and it is one of the first to make use of clinical trial evidence [14, 15]. This evaluation provides valuable evidence that can be used by decision makers to help assess the costs and benefits of alternative HCW mask/respirator protection strategies. The determination of cost-effectiveness will depend on the willingness to pay to prevent a CRI case in a HCW and this varies between countries and is not easily transferrable between different settings. The extent to which a decision maker is likely to focus on cost-effectiveness evidence when it comes to HCW protection will in part depend on the seriousness of the infections being prevented [33, 38]. In the case of a highly pathogenic pandemic, respirator use in HCWs would likely be a cost-effective intervention.

## Abbreviations

CDC: Centres for Disease Control and Prevention
CRI: Clinical respiratory illness
HCW: Healthcare worker
ICER: Incremental cost-effectiveness ratio
WHO: World Health Organization
